# Placental biomarkers of phthalate effects on mRNA transcription: application in epidemiologic research

**DOI:** 10.1186/1476-069X-8-20

**Published:** 2009-04-23

**Authors:** Jennifer J Adibi, Russ Hauser, Paige L Williams, Robin M Whyatt, Harshwardhan M Thaker, Heather Nelson, Robert Herrick, Hari K Bhat

**Affiliations:** 1 Department of Environmental Health, Harvard School of Public Health, Boston, MA, USA; 2 Department of Biostatistics, Harvard School of Public Health, Boston, MA, USA; 3 Department of Environmental Health Sciences, Mailman School of Public Health, New York, NY, USA; 4 Department of Pathology and Cell Biology, Columbia University, New York, NY, USA

## Abstract

**Background:**

CYP19 and PPARγ are two genes expressed in the placental trophoblast that are important to placental function and are disrupted by phthalate exposure in other cell types. Measurement of the mRNA of these two genes in human placental tissue by quantitative real-time polymerase chain reaction (qPCR) offers a source of potential biomarkers for use in epidemiologic research. We report on methodologic challenges to be considered in study design.

**Methods:**

We anonymously collected 10 full-term placentas and, for each, sampled placental villi at 12 sites in the chorionic plate representing the inner (closer to the cord insertion site) and outer regions. Each sample was analyzed for the expression of two candidate genes, aromatase (CYP19) and peroxisome proliferator activated receptor protein gamma (PPARγ) and three potential internal controls: cyclophilin (CYC), 18S rRNA (18S), and total RNA. Between and within placenta variability was estimated using variance component analysis. Associations of expression levels with sampling characteristics were estimated using mixed effects models.

**Results:**

We identified large within-placenta variability in both transcripts (>90% of total variance) that was minimized to <20% of total variance by using 18S as an internal control and by modelling the means by inner and outer regions. 18S rRNA was the most appropriate internal control based on within and between placenta variability estimates and low correlations of 18S mRNA with target gene mRNA. Gene expression did not differ significantly by delivery method. We observed decreases in the expression of both transcripts over the 25 minute period after delivery (CYP19 p-value for trend = 0.009 and PPARγ (p-value for trend = 0.002). Using histologic methods, we confirmed that our samples were comprised predominantly of villous tissue of the fetal placenta with minimal contamination of maternally derived cell types.

**Conclusion:**

qPCR-derived biomarkers of placental CYP19 and PPARγ gene expression show high within-placental variability. Sampling scheme, selection of an appropriate internal control and the timing of sample collection relative to delivery can be optimized to minimize within-placenta and other sources of underlying, non-etiologic variability.

## Background

The placenta is readily available yet underutilized in epidemiology as a biological marker of the effects of *in utero *exposures on the fetal/placental unit. As an endocrine organ, which plays a critical role in all aspects of pregnancy maintenance and fetal development, the placenta can provide insight into mechanisms by which specific exposures and risk factors are associated with outcomes measured at delivery. Outcomes which are placentally-mediated include the timing of labor [1], preeclampsia [2,3] intrauterine growth restriction [4,5], and possibly endocrine [6,7] and neurologic diseases [8] which develop later in life. In the present study, we explored the utility of placental gene expression measured by real-time quantitative polymerase chain reaction (qPCR) as a novel biomarker for use in environmental epidemiology, including in our research on prenatal exposures to common endocrine disrupting chemicals called phthalates.

Phthalates have been shown to be reproductive and developmental toxicants in animal models with some preliminary evidence of effects in humans [9,10]. Urinary phthalate metabolites can be characterized as low dose chronic exposures in >90% of pregnant women [11-13]. We chose to study the placenta as a potential target of phthalate toxicity during pregnancy. The placenta is a transient endocrine organ which assumes a wide range of functions to facilitate maternal-fetal interactions [14]. It supplants the ovary in the production, metabolism and regulation of steroid and other hormones necessary for pregnancy maintenance and fetal development [15]. The placenta, through autocrine and paracrine signaling, helps to maintain uterine quiescence until late pregnancy when a tightly regulated signaling cascade between the placenta, the fetus, and the uterus is initiated to stimulate uterine contractions [16]. Placental transporters can both block and facilitate xenobiotic entry into the fetal compartment [17].

The placenta is an extremely heterogeneous tissue and consists of several distinct cell populations. The main placenta cell type is the trophoblast which is fetal in origin and carries out most of the functions listed above. The maternal side of the placenta or basal plate emanates from the uterine wall and consists of trophoblastic and endometrium-derived cells. The fetal component or the placental villous is composed of a mixture of cell types including trophoblasts, the trophoblastic basement membrane, stroma (mesenchymal cells, macrophages, fibroblasts, and matrix components) and fetal vessels [18]. In the application of biomarkers of transcription, it is of critical importance to identify tissue type and cell type of interest and design methods to maximize the presence of these cells in any given biopsy. We were interested in targeting villous tissue and specifically gene targets expressed in the syncytiotrophoblast.

Existing, previously-validated methods for identifying toxic or pathologic responses in the human placenta include measuring the residues of the compound of interest in placental tissues [19-21]; documenting changes in morphology and histology as is done in routine pathological exams [22-24]; and looking at associations between the exposure and clinical cases of preeclampsia, placenta abruption and other clinical outcomes relevant to placental function [25-27]. DNA adducts have been measured in placental tissue and were associated with environmental exposures but not with enzyme activity, suggesting that they may not be good biomarkers of biochemical effects [17].

We were interested in identifying a biomarker that could be measured reliably and accurately in the placenta in an epidemiologic setting that was related to phthalate exposure and potentially relevant to effects at the molecular and clinical levels. Messenger RNA was chosen given the feasibility in measuring it in a large number of placentas and its clear physiologic relevance. qPCR was chosen over global gene expression microarrays, another commonly used method in studies using human placental tissue [28-32], for two reasons. We had identified our gene targets based on *apriori *evidence from the toxicologic literature. Secondly, qPCR was more cost-effective given that our ultimate goal was to analyze a large enough number of samples (N>150) to be able to detect associations with common, low dose environmental exposures.

This validation study was undertaken to quantify underlying variability for two gene targets that will be used in our epidemiologic research: CYP19 (aromatase) and PPARϒ (peroxisome proliferator receptor protein gamma). CYP19 codes for the enzyme aromatase that is responsible for the conversion of androstenedione to estradiol in the placenta. Estradiol is essential for fetal development and parturition signaling [15]. CYP19 may also be important in the metabolism of xenobiotic compounds [33,34]. PPARγ is a transcription factor that has been shown to play an essential role in placental development and function through the regulation of genes involved in trophoblast differentiation, angiogenesis, fatty acid transport, and inflammation [35]. PPARγ null mice die during embryogenesis due to gross placental malformation and cardiac defects in the mouse [36]. The expression of both CYP19 and PPARγ were altered in response to phthalate exposure in rodent models [37-42]. Within the chorionic villi, PPARγ and CYP19 expression are expressed primarily by trophoblasts. In term placentas, PPARγ is localized to a large degree to the syncytiotrophoblast [43,44]

The purpose to the study was four-fold: (1) to evaluate variability in gene expression by placenta, by quadrant and by inner and outer regions of the chorionic plate; (2) to determine which of three potential internal controls was most appropriate; (3) to evaluate the effects of sampling characteristics on mRNA levels such as delivery method and time elapsed from delivery to sample collection; and (4), given the extremely heterogeneous nature of placental tissue, to assess the cell type composition in our tissue biopsies using histologic methods. Due to the fact that this study was conducted on discarded tissue and exempt from human subject protection, we were not able to collect subject-specific information other than time of delivery and delivery method.

## Methods

**Figure 1:**
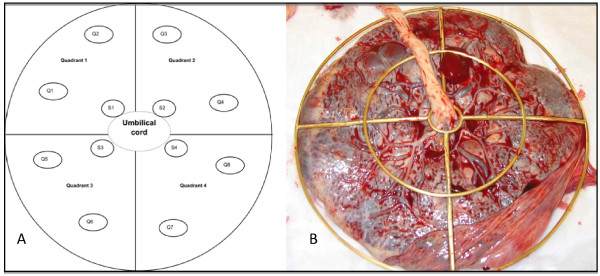
**Placental sampling scheme and device**. a) Samples S1 – S4 represent the "inner" region and samples Q1 – Q8 represent the "outer" region and b) Placenta is pictured looking down at the fetal side and the chorionic membrane. Sampling was carried out just below this membrane.

Placental Samples: Ten full-term, discarded placentas were collected in the labor and delivery room at Morgan Stanley Children's Hospital of New York Presbyterian. Placentas were collected anonymously and thus IRB (Institutional Review Board) exemption was granted by Harvard School of Public Health (HSPH) IRB and the Columbia Medical Center IRB. The placentas included Caesarian deliveries (C-section) (n = 3), vaginal normal deliveries (n = 3), vaginal induced deliveries (n = 3), and one vaginal augmented delivery (n = 1). Twelve tissue samples were collected per placenta according to the scheme in Figure 1a. A placental sampling device was used to orientate the fetal side of each placenta in relation to the umbilical cord (Figure 1b). The S1–S4 samples were categorized as 'inner' or from the area closest to the umbilical cord insertion. The Q1–Q8 samples were categorized as 'outer'. Care was taken by visual examination and dissection to minimize contamination from fetal membranes or maternal decidua and to maximize the amount of villous tissue in the sample. Each biopsy was taken approximately 1 – 1.5 cm below the fetal membrane to avoid membrane contamination as well as decidua contamination. The general dimension of each biopsy was 1–2 cubic centimeters and less than 1 gram in weight. Each sample was preserved in RNALater (Ambion, Austin, TX USA) to stabilize the RNA and stored at 4°C. Within 30 days, samples were transferred to -80°C. Samples were collected between 3 and 25 minutes after delivery (<= 10 minutes, n = 3; 11 – 20 minutes, n = 3; 21 – 25 minutes, n = 4). Samples were collected over one month in June – July 2005.

RNA Analysis: Total RNA was isolated from approximately 300 mg of tissue using the RNeasy-Midi Kit (Qiagen, Valencia, CA). Genomic DNA contamination in the sample was minimized with a DNase digestion step. Total RNA was measured by determining absorbance at 260 nm using an Ultrospec 2100 Pro UV/Visible Spectrophotometer (GE Healthcare, Piscataway, NJ USA). RNA purity was assessed by the A_260_/A_280 _ratio (mean = 1.8, SD = 0.13, n = 119). We also ran the isolated RNA samples on analytical agarose gels. If the gel showed clear bands for 28S and 18S, the samples were used for reverse transcription (RT). If not, we re-isolated the total RNA isolation from the same sample. Approximately 3 μg total RNA were used in a RT reaction to synthesize cDNA using the First Strand SuperScript from Invitrogen (Carlsbad, CA USA). Finally, qPCR was used to quantitate differences in mRNA levels in each sample for individual genes. Cyclophilin (CYC) and 18S ribosomal RNA (18S) were included as housekeeping genes to serve as internal controls for quantity and quality of cDNA going into the RT reaction. CYC was chosen based on a previous study that evaluated 3 housekeeping genes for use in human placental tissue analysis and found no difference between them [45]. 18S was later chosen as the consensus housekeeping gene based on the more recent placenta literature [30,32,46,47]. Total RNA (μg total RNA/sample) was also evaluated as a potential internal control [48,49]. All samples were analyzed for PPARγ, CYP19, CYC and 18S mRNA using the ABI Prism 7500 Sequence Detection System (Applied Biosystems, Foster City, CA USA). Cycling conditions were the same for all four transcripts: 95.0 C for 5:00 minutes for activation of the enzyme, 95.0 C for 30 seconds for denaturation and 60.0 C for 1:00 minute for annealing/extension for 40 cycles, followed by a dissociation step. Forward and reverse primers (Sigma, St. Louis, MO USA) were either designed by Primer 3 [50] or selected for each gene to maximize specificity and efficiency in the reaction: CYP19 (249 base pairs (BP)) (forward) 5'-ATACCAGGTCCTGGCTACTG-3' and CYP19 (reverse) 5'-TCTCATGCATACCGATGCACTG-3'[51]; PPARγ (225 BP) (forward) 5'-GCTGTGCAGGAGATCACAGA-3' and PPARγ (reverse) 5'-GGGCTCCATAAAGTCACCAA-3'; CYC (116 BP) (forward) 5'-CCCACCGTGTTCTTCGACAT-3' and CYC (reverse) 5'-CCAGTGCTCAGAGCACGAAA-3'[52]; and 18S (forward) 5'-CGGCTACCACATCCAAGGAA-3' and 18S (187 BP) (reverse) 5'-GCTGGAATTACCGCGGCT-3' [53]. Each reaction used 2 μl cDNA, forward and reverse primers at optimized concentrations, and SYBR Green PCR Core Reagents kit for a total reaction volume of 25 μl.

Specificity and Quantitation: Each sample was run in duplicate. The duplicate values not falling within 50% of their mean were rerun. Specificity of the PCR product was evaluated using the melting curve generated at the end of amplification and by running a 2% agarose gel to visualize the PCR product. Absolute quantitation of mRNA concentration in the original sample was achieved using a standard curve generated for each batch [54]. Each standard curve included 2 non-template controls and 8 serial dilutions covering the range of 1000 molecules/μl – 1 × 10^7 ^molecules/μl. The standards for each gene were prepared as described previously [55]. The R^2 ^for the standard curve was between 0.98–1.00. The plate was rerun if it fell below 0.95. Ct (threshold cycle) values were use to evaluate batch effects. These are the raw data generated by the qPCR and which are used in combination with the standard curve and the internal control to calculate the number of mRNA molecules in each sample. The number of target gene mRNA molecules for each sample was divided by the number of CYC or 18S molecules or μg total RNA for that same sample and expressed as a unitless ratio.

Histologic confirmation: Histologic confirmation of cell type composition was done by using a subset of 5 representative placentas selected from the full set of 10 placentas used for qPCR. From each placenta, a piece of tissue was cut from an inner sample (closer to umbilical insertion point) and an outer sample (closer to outer margin of the chorionic plate), treated with RNA-Later and stored long-term at -80 degrees Celsius. Later they were rinsed and fixed in formalin. They were then paraffin embedded, cut and stained with hematoxylin and eosin and examined by a placental pathologist.

Statistical analysis: Gene expression values were transformed to more closely approximate a normal distribution; for CYP19/CYC and CYP19/total RNA, we were able to approximate normality with a square root transformation and for all others we used a natural log transformation. One CYP19 value for placenta ID 2 and sample Q7 was extremely low (<2 molecules mRNA/sample). We concluded that the original mRNA concentration was below detection and excluded it from the analysis. Two tissue samples were missing completely and three samples were missing 18S values, reducing sample size from N (placentas) = 10 and n (tissues samples) = 120 to N = 10 and n = 115. The distributions of the untransformed values were summarized using means per placenta. Spearman correlation coefficients were used to evaluate correlations between genes within samples. Mixed effects models were used to estimate differences in gene expression by delivery method and time elapsed since delivery, and regional (inner versus outer placenta) differences. The correlation structure assumed equal variance between any 2 of the 12 samples. Variance component analysis was used to estimate between-placenta versus within-placenta variance. Statistical significance was set at p-value equal to or less than 0.05. SAS 9.1 (SAS Institute, Cary, NC USA) software was used to conduct all analyses.

## Results and discussion

### Histologic confirmation of cell type

**Figure 2:**
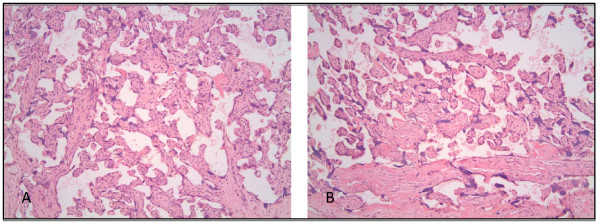
**Histologic confirmation of cell type composition done with H&E stained cross-sections of RNA-Later treated placental biopsies**. a) inner region of a vaginally-delivered placenta and b) outer region of a vaginally-delivered placenta.

The histologic analysis of RNA-Later treated sample confirmed that the samples were composed of placental villous tissue, with normal architecture and branching of the villi (Figure 2). There were no pathological changes, such as infarcts, hematomas, thrombi or inflammation in any of the 5 placentas evaluated. The syncytiotrophoblast was present in the expected proportion to the remainder of the cell types. Samples did not contain chorionic membrane. Decidual cells made up approximately 5% of the total nuclei based on what was viewed on the slide. We did not see any obvious differences in the histology or cell type composition between the inner and outer samples, or by quadrant.

### Distribution of total RNA and gene expression values

Each tissue biopsy yielded on average 113 μg total RNA/sample (standard deviation (SD) = 108, n = 119). The mean starting weight of the samples was 191 mg (SD 57). The median mRNA concentrations were highest for 18S (1.5 × 10^6 ^mRNA molecules/sample), then CYP19 (9.8 × 10^5^), CYC (4.4 × 10^5^) and lowest for PPARϒ (1.0 × 10^4^). The coefficients of variation for the batch Ct values were all less than 10% (3% for PPARγ and CYC, 6% for CYP19, and 9% for 18S).

### Between vs. within placenta variability

**Figure 3:**
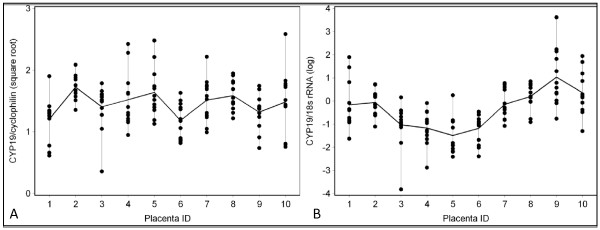
**Raw data shows high within-placenta variation in gene expression that is highest when cyclophilin is used as an internal control compared to 18S**. a) CYP19 mRNA adjusted for CYC mRNA b). CYP19 mRNA adjusted for 18S mRNA. The vertical lines and black dots represent values for individual biopsies within a placenta. The gray lines connect the median values per placenta.

**Figure 4:**
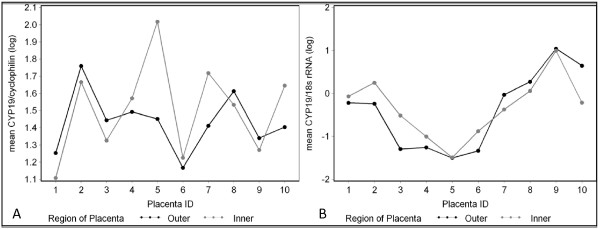
**The means by inner and outer region show less within-placenta variation**. a) mean CYP19 mRNA adjusted for CYC mRNA b) mean CYP19 mRNA adjusted for 18S mRNA. Black points represent the outer region and gray represents the inner region.

Within-placenta variability was greater than between-placenta variability for the 10 placentas when we used all 12 samples per placenta, ranging from 54 to 99% of the total variation (Table 1 and Figure 3). The ratio of between to within placenta variability also varied with different internal controls as seen in the comparison of Figures 3a vs. 3b and Figures 4a vs. 4b. Adjustment for CYC as an internal control produced the most extreme estimates in the ratio (>90% within-placenta variation of total variance). When we modelled the means by the 4 quadrants, the within-placenta variability decreased to 50% or less of the total variability when values were adjusted for 18S and total RNA (Table 1). The means in CYP19 values by inner and outer region of the chorionic plate (black and gray dots) were closely aligned when we adjusted for 18S (<20% within-placenta variability) (Table 1, Figure 4b) and less closely aligned when we adjusted for cyclophilin (57% within-placenta variability) (Table 1, Figure 4a).

**Table 1:** Variance component analysis.

	a) Cyclophilin		b) 18S rRNA		c) total RNA	
	PPARγ	CYP19	PPARγ	CYP19	PPARγ	CYP19

**By sample number (n = 12/placenta)**						

Between	1%	8%	36%	46%	26%	37%

Within	99%	92%	64%	54%	74%	63%

**By quadrant (n = 4/placenta)**						

Between	9%	14%	53%	67%	50%	66%

Within	91%	86%	47%	33%	50%	34%

**By region (n = 2/placenta)**						

Between	0%	43%	81%	84%	49%	77%

Within	100%	57%	19%	16%	51%	23%

Results vary when the target mRNA is adjusted by a) CYC; b) 18S rRNA; and c) total RNA.

### Spatial variability

Samples taken from the inner region (area closer to the cord insertion point as depicted in Figure 1a) had higher PPARγ expression than samples taken further from the cord insertion point; although not statistically significant. There was no difference by region in CYP19 expression (Table 2). We did not include quadrant in the mixed effects models as they were intended to provide structure to the sampling scheme. The quadrants do not hold anatomic or physiologic significance.

**Table 2:** Sample collection characteristics as multivariate predictors of gene expression.

	CYP19/18S (square root)	PPARγ/18S (log)
**Mean (SD) by placenta**	-0.34 (1.1)	-4.70 (1.4)

**Characteristic**	β **coefficient (SE) p-value**	

**Delivery method**		

Vaginal vs. c-section	0.28 (0.39) p = 0.50	0.52 (0.31) p = 0.15

**Time Elapsed since delivery up to 30 minutes (<= 10 minutes is referent)**		

11 – 20 minutes	-0.82 (0.39) p = 0.08	-1.24 (0.32) p = 0.008

21 – 30 minutes	-1.99 (0.30) p = 0.0005	-2.12 (0.40) p = 0.002

*p-value for trend*	0.009	0.002

**Regional differences**		

Inner vs. outer	0.04 (0.15) p = 0.77	0.20 (0.18) p = 0.26

Tissue heterogeneity as well as differences in perfusion and the degree of hypoxia are major sources of within-placenta variability [31,45,46]. Wyatt et al. did a detailed analysis of concordance between histologic parameters and gene expression using 6 placentas sampled at 9 sites including the chorionic (fetal side) and basal (maternal side) plates and the inner (medial) and outer (lateral) regions on both sides [46]. They observed evidence of higher expression of hypoxia-related placental transcripts in the areas with histology characteristic of lower perfusion, i.e. small villi, increased amounts of fibrin deposits, and common syncytial knots. They concluded that perfusion was highest in the maternal-side inner samples and the lowest in the fetal-side outer samples. Consistent with differences by degree of hypoxia, we also saw a non-significant trend towards higher expression of the hypoxia-related transcript PPARγ in the inner region. PPARγ is a regulator of the response to hypoxia in the trophoblast [56]. Our measure of the expression of the gene for the receptor gene could be directly or inversely correlated with increased hypoxia-induced ligand-receptor interaction depending on the types of feedback mechanisms involved.

### Normalization strategies: cyclophilin vs. 18S rRNA vs. total RNA

Using unadjusted values, CYP19 was weakly but significantly correlated with the internal controls: CYC (r = 0.34), 18S (r = 0.30), and total RNA (r = 0.19). PPARγ was strongly correlated with CYC (r = 0.53), not correlated with 18S (r = 0.05, p = 0.64), and weakly correlated with total RNA (r = 0.18 p = 0.05).

The CYP19 – PPARγ correlation was significant regardless of internal control and strongest with 18S adjustment (r = 0.85), also strong with total RNA adjustment (r = 0.64), and weakest with CYC adjustment (r = 0.25). There was no overlap in the 95% confidence intervals for these three correlation coefficients, suggesting that there are significant differences in the point estimate depending on which internal control is used. PPARγ is a potential regulator of CYP19 transcription in the trophoblast given its known relationship in the granulosa cell [57] and therefore we would expect their expression values to be correlated. If we used CYC as an internal control, we would have erroneously concluded that CYP19 and PPARγ expression were not correlated based on the considerably lower r-value than when we used 18S.

Adjustment for an appropriate internal control should in theory minimize variability due to differences in the quality and quantity of the RNA that went into the RT reaction. Several possible internal controls have been proposed for qPCR including total RNA, ribosomal RNAs, and one or more reference messenger RNAs [48,49,58]. Reference mRNAs are the most common approach. Some of the criteria are that they be constitutively expressed in the cell type of interest, in the tissue of interest and not be affected by the exposure of interest or experimental conditions. The internal control should not share regulatory factors with the gene of interest. It should have low intra-individual variation and should be expressed at roughly the same order of magnitude as the target gene [49,59].

Unlike Pidoux et al. (2004) who showed that cyclophilin was a suitable housekeeping gene in studies of PPARγ expression in the trophoblast, we found that cyclophilin was not tenable as a housekeeping gene in our conditions. It showed extremely high within-placenta variability that we speculate is related to its role in hypoxia therefore obviating its use as a housekeeping gene. Reports of CYC expression in other tissues showed that it was upregulated in response to hypoxia which would make it inappropriate for use in placental studies where hypoxia might be in the causal pathway between exposure and disease [60]. We also saw that CYC mRNA was correlated with PPARγ mRNA suggesting that they might share one or more regulatory factors. Previous reports have shown that PPARγ expression in the placenta is partly regulated by hypoxia [61,62]

We evaluated total RNA as a potential internal control. Total RNA is thought to vary minimally by cell and therefore be more stable within a tissue type, to be less variable by method of homogenization, more uniformly measured across studies, and possibly a more accurate measure of the quality of the tissue at the time of sampling [49,59]. We found that total RNA did better than CYC in controlling for within-placenta variation. However, it is problematic in that it does not take into account differences in reverse transcription efficiencies by which the synthesized cDNA may or may not be directly proportional to the amount of input total RNA [48,49,59]. To our knowledge, there are no published placenta studies that have used total RNA as an internal control.

Ribosomal RNAs such as 18S have been recommended as reference genes in that they are generated by a polymerase distinct from those that generate mRNA, they are less likely to vary by conditions that affect the expression of mRNA, and they have been shown to be more reliable than other housekeeping genes in a variety of tissues [59]. 18S appears to be the most commonly used reference gene in placental studies of gene expression [30,32,46,47]. Additional reference genes (SDHA, TBP and YWHAZ) have recently been proposed for placenta studies that are expressed are lower levels than 18S and should be considered in future studies [58,63].

### Effects of sampling characteristics on gene expression

Lastly, we were interested in estimating to what degree sampling characteristics predicted variability in the target gene expression. We fit multivariate mixed effects models that simultaneously adjusted for delivery method, time elapsed from delivery and region (inner vs. outer) (Table 2). Target mRNA levels are adjusted for 18S. PPARγ expression was higher in vaginal deliveries although not statistically significant. There was no difference in CYP19 expression by delivery method.

All placentas were sampled within 25 minutes after delivery. 67% of C-section placentas were collected within 10 minutes of delivery vs. 14% of the vaginally-delivered placentas. Expression levels decreased with time from delivery to collection which could be due to tissue necrosis, lost of oxygen supply, changes in temperature, handling of tissue, etc. We observed significant decreases over 25 minutes in the expression of both transcripts (Table 2). We observed a trend towards higher PPARγ expression in laboring placentas which is consistent with reports that PPARγ plays a role in the production of uterine contractions through the regulation of cytokine and prostaglandin production [64,65].

A common practice in clinical and basic research settings is to restrict to C-section placentas due to tighter control over the time of sampling relative to the birth of the baby. In a setting, i.e. prenatal phthalate exposure [66], where the exposure of interest may have a putative effect on parturition signalling, this type of exclusion may introduce bias and possibly preclude the detection of associations of interest. For this reason, we propose sampling placentas of all delivery methods and routinely recording detailed information on the method and conditions of labor and delivery to be used in the data analysis. Likewise, we recommend evaluating the impact of the time elapsed from delivery to collection on expression values of specific transcripts.

All of the placentas in this study were full-term. However, because the tissue collection was IRB-exempt we were not able to collect information on specific gestational age which has been shown to be associated with placental gene expression [32]. Other variables that would have been important in characterizing underlying variability in these transcripts are sex of the baby given that CYP19 expression is known to be sexually dimorphic [67,68], and ethnicity of the mother and father to control for know polymorphisms [69,70]. Lack of control for these variables could have resulted in bias in the associations that we report here. Technician and batch effects could also be important. We did not see evidence of batch effects in this analysis based on Ct values and one researcher carried all of the sampling and analysis out over a short period. It would be informative in a future validation study to correlate CYP19 and PPARγ protein levels to mRNA levels in order to better understand the phenotypic significance of transcriptional markers. Messenger RNA gives us information on effects in gene regulation solely at the level of transcription, which is an important yet singular piece of a complex process.

## Conclusion

In a small study to validate methods for measuring CYP19 and PPARγ placental gene expression for application in an epidemiologic study, we identified methodologic challenges which we were able to partly resolve for these two transcripts. The application of placental biomarkers of transcription in epidemiologic studies will allow for the testing of a wide range of hypotheses relating to environmental hazards in pregnancy and their potential role in fetal origins of disease. The primary challenge was developing a reproducible sampling scheme that can give a representative snapshot of gene expression for a given placenta while not being overpowered by the high within-placenta variability in these two transcripts. In an epidemiologic analysis, the statistical ability to identify predictors of gene expression can be greatly diminished as the ratio of within placenta variance/total variance increases much over 50%.

Another challenge was the selection of an appropriate internal control that represents mRNA levels in the cell at the time of sampling independent of factors regulating the target mRNA. Thirdly, given that the placenta is a highly dynamic tissue that reaches its physiologic climax immediately prior to being sampled, characteristics such as delivery method and time elapsed from delivery can potentially introduce unwanted variability. As our goal was to measure gene expression in the fully differentiated endocrine cell type, the syncytiotrophoblast, we included an additional step to confirm that this cell type was present.

Based on the results of this study, we recommend sampling from different regions within the area of interest (i.e. fetal or maternal side) to capture physiologically relevant spatial variability. In order to target trophoblast expression, we devised an efficient design by sampling the inner and outer regions of the fetal side or chorionic plate. If more than two samples are collected, mean values for the inner and outer regions may be modelled as the dependent variable. Methods can be further tailored to reduce the impact of variability within a placenta when the objective is to compare gene expression profiles between placentas. Although, we cannot recommend a universally appropriate internal control for placental studies, 18S seems to give the least within-placenta variation for CYP19 and PPARγ.

## List of abbreviations

CYP19: aromatase; PPARγ: peroxisome proliferator activated receptor gamma; qPCR: quantitative real-time polymerase chain reaction; CYC: cyclophilin; 18S: 18S ribosomal RNA; IRB: Institutional Review Board; SD: standard deviation.

## Competing interests

The authors declare that they have no competing interests.

## Authors' contributions

JA conducted the placenta sampling, all lab analyses, data analysis and drafted the manuscript. HK provided mentorship and expertise in qPCR. RH, PW, RW, HN, RH and HK contributed to study design, data analysis and manuscript preparation. HT conducted the histologic analysis.
